# The METEX study: Methotrexate versus expectant management in women with ectopic pregnancy: A randomised controlled trial

**DOI:** 10.1186/1472-6874-8-10

**Published:** 2008-06-19

**Authors:** Norah M van Mello, Femke Mol, Albert H Adriaanse, Erik A Boss, Antonius B Dijkman, Johannes PR Doornbos, Mark Hans Emanuel, Jaap Friederich, Loes van der Leeuw-Harmsen, Jos P Lips, Evert JP van Santbrink, Harold R Verhoeve, Harry Visser, Willem M Ankum, Fulco van der Veen, Ben W Mol, Petra J Hajenius

**Affiliations:** 1 Department of Obstetrics and Gynaecology, Academic Medical Centre, University of Amsterdam, Amsterdam, The Netherlands; 2 Department of Obstetrics and Gynaecology, Medical Centre Alkmaar, Alkmaar, The Netherlands; 3 Department of Obstetrics and Gynaecology, Maxima Medical Centre, Veldhoven, The Netherlands; 4 Department of Obstetrics and Gynaecology, Boven IJ Hospital, Amsterdam, The Netherlands; 5 Department of Obstetrics and Gynaecology, Zaans Medical Centre, Zaandam, The Netherlands; 6 Department of Obstetrics and Gynaecology, Spaarne Hospital, Hoofddorp, The Netherlands; 7 Department of Obstetrics and Gynaecology, Gemini Hospital, Den Helder, The Netherlands; 8 Department of Obstetrics and Gynaecology, Deventer Hospital, Deventer, The Netherlands; 9 Department of Obstetrics and Gynaecology, Kennemer Gasthuis, Haarlem, The Netherlands; 10 Department of Obstetrics and Gynaecology, Erasmus Medical Centre, Rotterdam, The Netherlands; 11 Department of Obstetrics and Gynaecology, Onze Lieve Vrouwe Gasthuis, Amsterdam, The Netherlands; 12 Department of Obstetrics and Gynaecology, Tergooi Hospital, Blaricum, The Netherlands; 13 Centre for Reproductive Medicine, Academic Medical Centre, University of Amsterdam, Amsterdam, The Netherlands

## Abstract

**Background:**

Patients with ectopic pregnancy (EP) and low serum hCG concentrations and women with a pregnancy of unknown location (PUL) and plateauing serum hCG levels are commonly treated with systemic methotrexate (MTX). However, there is no evidence that treatment in these particular subgroups of women is necessary as many of these early EPs may resolve spontaneously. The aim of this study is whether expectant management in women with EP or PUL and with low but plateauing serum hCG concentrations is an alternative to MTX treatment in terms of treatment success, future pregnancy, health related quality of life and costs.

**Methods/Design:**

A multicentre randomised controlled trial in The Netherlands. Hemodynamically stable patients with an EP visible on transvaginal ultrasound and a plateauing serum hCG concentration < 1,500 IU/L or with a persisting PUL with plateauing serum hCG concentrations < 2,000 IU/L are eligible for the trial. Patients with a viable EP, signs of tubal rupture/abdominal bleeding, or a contra-indication for MTX will not be included. Expectant management is compared with systemic MTX in a single dose intramuscular regimen (1 mg/kg) in an outpatient setting. Serum hCG levels are monitored weekly; in case of inadequately declining, systemic MTX is installed or continued. In case of hemodynamic instability and/or signs of tubal rupture, surgery is performed. The primary outcome measure is an uneventful decline of serum hCG to an undetectable level by the initial intervention. Secondary outcomes are (re)interventions (additional systemic MTX injections and/or surgery), treatment complications, health related quality of life, financial costs, and future fertility. Analysis is performed according to the intention to treat principle. Quality of life is assessed by questionnaires before and at three time points after randomisation. Costs are expressed as direct costs with data on costs and used resources in the participating centres. Fertility is assessed by questionnaires after 6, 12, 18 and 24 months. Patients' preferences will be assessed using a discrete choice experiment.

**Discussion:**

This trial will provide guidance on the present management dilemmas in women with EPs and PULs with low and plateauing serum hCG concentrations.

**Trial registration:**

Current Controlled Trials ISRCTN 48210491

## Background

In industrialized countries the incidence of ectopic pregnancy (EP) is approximately 1 to 2% of all pregnancies [1-4]. EP is usually diagnosed by non-invasive methods, i.e. by sensitive pregnancy tests in urine and serum, and high resolution transvaginal sonography (TVS), which have been integrated in reliable diagnostic algorithms. These algorithms, in combination with increased awareness and knowledge of risk factors among both clinicians and patients, have enabled an early and accurate diagnosis of EP [5-7].

As a consequence, the clinical presentation of EP has changed from a life threatening disease, necessitating emergency surgery, to a more benign condition in frequently asymptomatic patients for whom non-surgical treatment options are available, i.e. medical treatment with systemic methotrexate (MTX) treatment or expectant management.

MTX can be administered systemically in a multiple dose regimen (MTX 1.0 mg/kg intramuscularly (im) day 0, 2, 4, 6, alternated with folinic acid 0.1 mg/kg orally day 1, 3, 5, 7) or in a single dose regimen (MTX 1.0 mg/kg or 50 mg/m^2 ^im without folinic acid) [8,9]. A single dose regimen was introduced to minimize side effects, to improve patients' compliance and to reduce overall costs. MTX has been shown to be safe with virtually no adverse effects reported on reproductive outcome [10]. Data provided by randomised controlled trials (RCTs) indicate that systemic MTX treatment should only be used in selected patients with EP. Important selection criteria are the EP size, absence of fetal cardiac activity on TVS, and maximum human chorionic gonadotrophin (hCG) concentrations [11,12].

Expectant management has been practiced based on the acknowledgement that the natural course of many early EPs is a self limiting process, ultimately resulting in tubal abortion or reabsorption. To date, expectant management has not been properly evaluated in RCTs in selected patients with EP [12].

In these non-surgical treatments, the pregnancy is not removed and as a consequence there is a remaining risk of tubal rupture. Therefore, intensive serum hCG monitoring is mandatory to detect impending treatment failure and/or inadequately declining serum hCG concentrations [13,14]. The risk of tubal rupture combined with the need for meticulous follow-up is likely to cause distress in the patient because of uncertainty about treatment outcome [15]. Non-surgical treatment modalities may therefore have a negative impact on the patients' health related quality of life.

In some women presenting with suspected EP, the pregnancy cannot be identified on TVS [16,17]. These women with a so called pregnancy of unknown location (PUL) can be managed expectantly with monitoring of serum hCG to identify whether a PUL turns out to be an intra uterine pregnancy (IUP), an EP, a failing PUL with an uneventful serum hCG decline to undetectable levels, or a persisting PUL with plateauing serum hCG concentrations [18].

Women with a visible EP but with low and plateauing serum hCG concentrations and women with a persisting PUL have thus far been offered medical treatment with MTX [17,19]. However, there is no evidence on the effectiveness of MTX as compared to expectant management. This study compares both treatment options in these particular subgroups of women, representing about 10% of women with suspected EP.

## Objective

To study whether expectant management is an alternative to treatment with systemic MTX in a single dose im regimen in women with an EP and low but plateauing serum hCG concentrations in terms of tubal rupture, future pregnancy, health related quality of life and costs.

## Methods

### Participating centres

This study is a multicentre randomised controlled trial in The Netherlands and inclusion started in April, 2007.

### Inclusion criteria

Hemodynamically stable patients ≥ 18 years of age, with either a visible EP on TVS (an ectopic ring or an ectopic mass and/or fluid in the pouch of Douglas) together with plateauing serum hCG concentrations < 1,500 IU/L, and those women with a PUL in combination with plateauing serum hCG concentrations < 2,000 IU/L, are eligible for the trial. The difference in serum hCG cut-off levels for these two entities is based on our previous studies [5,7]. Patients with a viable EP, signs of tubal rupture, active intra abdominal bleeding or a contra indication for MTX (e.g. abnormalities in liver or renal function or at full blood count) are not included.

### Ethical considerations

Approval for this study was obtained from the Medical Ethical Committee of the Academic Medical Centre and from the Central Committee on Research involving Human Subjects (CCMO), The Netherlands. A quality assessment has been made and approved by three external referees, experts from the field by the Netherlands Organization for Health Research and Development (ZonMw). A blinded interim analysis will be performed halfway the study by the Data Monitoring and Safety Committee. In patients fulfilling the inclusion criteria, written informed consent is obtained before randomisation is carried out. Women refusing participation are registered.

### Randomisation

Randomisation is performed by a web-based randomisation program, using a computer program with stratification for hospital and serum hCG concentration (< 1,000 IU/l versus 1,000–2,000 IU/l).

### Interventions

Expectantly managed women will get no specific treatment.

Women in the group allocated to MTX are administered a single MTX injection, 1 mg/kg body weight im, within 24 hours after randomisation.

**Figure 1:**
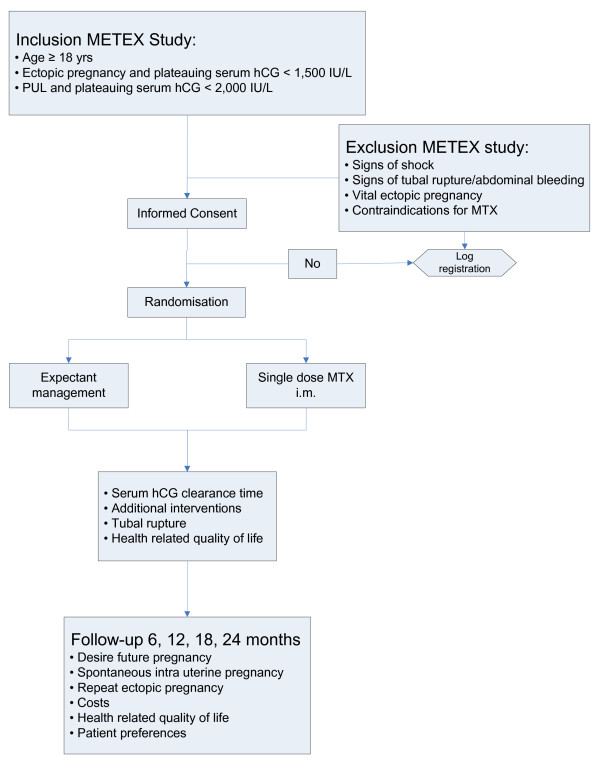
Flowchart METEX study.

All Rhesus negative women will receive 375 IE anti D im. For pain relief, if necessary, Paracetamol is prescribed. Patients are advised to refrain from sexual intercourse until serum hCG is undetectable. Treatment and follow up will be carried out in the outpatient clinic (Figure 1).

### Follow up

#### Short term follow up

In both groups weekly serum hCG measurements will be performed until serum hCG is no longer detectable. Serum hCG concentrations are expressed in IU/L (conversion factor to SI unit, 1.00 according to the World Health Organization Third International Standard 75/537). Seven days after a MTX injection liver and renal function and full blood count are checked. Complications will be registered in the Case Record Form.

In patients treated expectantly, treatment with systemic MTX (single shot 1 mg/kg im) is started whenever at any of the weekly follow up visits the serum hCG concentration has risen > 15% of the prior value. Expectant management is continued if the serum hCG concentration falls by > 15% of the prior value [20]. In case of a persistent plateauing serum hCG concentration, defined as < 15% fall or < 15% rise, the serum hCG concentration is assessed after 48 hours to ensure it is not increasing. If it is increasing as described above, treatment with systemic MTX as described above is installed. Whenever hemodynamic instability and/or clinical signs of tubal rupture (i.e., increasing abdominal pain in combination with falling haemoglobin level and signs of intra abdominal haemorrhage on TVS) occur, surgical intervention is carried out.

In patients treated with MTX, an additional MTX injection is given in case the serum hCG concentration on day 7 has declined <15% of the initial value on day 1 (start of treatment) [11]. If the serum hCG concentration fails to fall by at least 15% during any successive week of follow-up, repeated doses of MTX are given, with a maximum of three additional injections [21]. In case of hemodynamic instability and/or signs of tubal rupture, i.e., increasing abdominal pain in combination with falling haemoglobin level and signs of intra abdominal haemorrhage on TVS, or whenever more than four MTX injections are required, surgical treatment is indicated.

#### Long term follow up

Women who have been treated with MTX are advised not to get pregnant within three months after treatment [18]. To assess fertility in both treatment arms, patients are contacted every six months for a period of 24 months, by means of questionnaires. Questions focus on the desire for pregnancy, unprotected sexual intercourse with a chance of spontaneous conception, contraceptive use, infertility treatment, and the occurrence of any pregnancies and their outcomes.

### Outcome measures

#### Primary outcome measure

The primary outcome measure is an uneventful decline of serum hCG to an undetectable level (< 2 IU/l) by the initial intervention strategy, i.e. expectant management or a single dose systemic MTX.

#### Secondary outcome measures

Secondary outcome measures are (re)interventions (additional MTX injections or surgical procedures for persistent trophoblast and/or clinical signs), treatment complications, health related quality of life, financial costs, and future fertility. Moreover, patients' preferences will also be assessed.

Health related quality of life is assessed by standard self administered psychometric questionnaires with established viability and reliability at different time points, i.e. before randomisation, after one week, four weeks and three months.

Costs are expressed in direct costs with data on costs and used resources in the participating centres.

Future fertility is defined as time to the occurrence of a spontaneous viable IUP. A viable IUP is defined as the presence of fetal cardiac activity at a gestational age of ≥ 12 weeks. If an IUP does not occur, follow-up ends at the last consultation. In addition to IUP, repeat EP is also assessed. The date of occurrence of an EP will be determined from the first day of the last menstrual period.

Patients' preferences are assessed by an online questionnaire using a discrete choice experiment (DCE) based on characteristics of both interventions and will be compared with a control group, recruited among women visiting the infertility clinics of the participating hospitals.

#### Analysis

Data analysis is performed according to the intention to treat principle.

Short term outcome measures are compared by calculating relative risks and their 95% confidence intervals. Future fertility is assessed by life table analysis. Kaplan-Meier curves are constructed, estimating the cumulative probability of time to spontaneous IUP and repeat EP. If a spontaneous viable IUP does not occur, follow up ends at the last date of consultation, or at the start of in vitro fertilisation (IVF) treatment or the date of tubal surgery. Spontaneous conceptions that occur after failed IVF treatment will be registered. The log-rank test is used to test differences between the Kaplan-Meier curves for statistical significance. The differences between both treatment modalities are expressed as a fecundity rate ratio with 95% confidence interval, calculated through Cox proportional hazard analysis.

Changes in health related quality of life over time, and differences between the two groups will be measured using analysis of variance.

Depending on differences of equivalence between the strategies, the economic evaluation will be a cost-effectiveness analysis or a cost-minimisation analysis.

Patient's preferences are analysed by differences in outcome of the DCE.

#### Power calculation

Assuming an uneventful decline of serum hCG of 60% in the expectant management group and of 90% in the MTX group, and assuming a power of 80% and a significance level of 5%, 36 patients in each group are needed [6,15,22-24].

## Discussion

About 10% of women presenting with suspected EP, are diagnosed with a visible EP but with low and plateauing serum hCG concentrations or a persisting PUL [24]. Thus far, these particular subgroups of women are offered medical treatment with MTX based on the proven effectiveness of systemic MTX in women with selected EPs [12,16,19]. The question is whether it is really necessary to treat these women at all. This RCT will provide evidence whether expectant management is an alternative to systemic MTX in a single dose im regimen in women with an EP with low but plateauing serum hCG concentrations or persisting PUL.

Simultaneously with our trial, another trial is ongoing in the United Kingdom [20]. This placebo controlled trial has a similar objective, uses the same inclusion criteria and interventions (single shot MTX im and expectant management). In the UK trial, however, surgical intervention is installed in both treatment groups if inadequately declining serum hCG concentrations occur after one week of follow up. In our study, these treatment failures are treated with additional MTX injections. In the near future, meta analysis of the results of both trials will provide guidance on the present management dilemmas in women with EP or PUL with low and plateauing serum hCG concentrations.

## Competing interests

The authors declare that they have no competing interests.

## Authors' contributions

NMvM is responsible for the overall logistical aspects of the trial and drafted the paper. PJH designed the trial, was responsible for the development of the protocol, applied for a grant and has overall responsibility for the trial. FM contributed to the development of the protocol and commented on the draft paper. WMA, FvdV and BWM contributed to the development of the protocol, assisted in grant application, and commented on the draft paper. AHA, EAB, ABD, JPRD, MHE, JF, LvdL–H, JPJ, EJPvS, HRV and HV are responsible for the local logistical aspects of the trial and commented on the draft paper. All authors read and approved the final paper.

## Pre-publication history

The pre-publication history for this paper can be accessed here:


